# Study on the Expression Differences and the Correlation with H2BE Gene of Th Related Cytokines in SSDHS and LDSDS TCM-Syndromes of CHB Patients

**DOI:** 10.1155/2021/6291428

**Published:** 2021-02-22

**Authors:** Chao Liu, Yanfeng Zheng, Xia Li, Baixue Li, Li Wen, Dong Wang, Quansheng Feng, Cen Jiang

**Affiliations:** College of Basic Medical Sciences, Chengdu University of Traditional Chinese Medicine, Chengdu 610075, China

## Abstract

Although our previous studies revealed that H2BE exhibited significantly differential expression between two CHB TCM-syndromes: Spleen-stomach dampness-heat syndrome, SSDHS and liver-depression spleen-deficiency syndrome, LDSDS, the underlying mechanisms remain largely unknown. Recent studies showed that dynamic expression fluctuation of Th related cytokines in CHB TCM-syndromes, and furthermore, their expression levels were largely regulated by H2BE. This study aims to detect the expression level differences of Th related cytokines between these two TCM-syndromes and further investigate the underlying regulatory mechanisms. The expression levels of the four Th related cytokines and H2BE were analyzed and the protein-protein interaction networks between H2BE and the four cytokines were constructed. Our results suggested that almost all the cytokines were significantly upregulated compared with the healthy group (*P* < 0.05). Interestingly, among the four cytokines, only IL-4 and INF-*γ* showed statistical significance between these two syndromes. The protein-protein interaction networks demonstrated that H2BE was indirectly associated with IL-4 and IL-10, and H2BE may regulate the expression levels of cytokines through GATA3. Taken together, our results indicated that IL-4 and INF-*γ* are two representative cytokines that may serve as two potential biochemical indicators of SSDHS and LDSDS in CHB patients; except what has been reported, our study found that one possible way for H2BE to regulate the expression of cytokines is to interact with GATA3 directly or indirectly.

## 1. Introduction

Chronic hepatitis B (CHB) is a serious worldwide health problem that is often tightly associated with serious liver diseases, such as liver cirrhosis and even hepatocellular carcinoma (HCC) [1]. It was reported that nearly 20–30% of chronic hepatitis patients could further lead to cirrhosis or HCC due to the failure of virus clearance, and 15–40% of chronic patients will progress to cirrhosis and even HCC without any treatments [2, 3]. Globally, around 257 million people are suffering HBV, and sexual, blood and perinatal are the three major modes of HBV transmission [4, 5]. It is worth noting that among the three transmission modes, vertically from mother to child is the major cause for chronic HBV infection which account for 80–90% of cases of chronic HBV infection, and nearly 5–10% cases were infected during adolescence and adulthood [1, 6]. Therefore, CHB has become a serious disease affecting human health.

For CHB treatment, the goal is to improve the life quality and prevent the formation of cirrhosis and HCC, and unfortunately, there is still no specific medicine that could cure it completely in western medicine. Chinese Traditional Medicine (TCM), as complementary and alternative medicine, is becoming more and more popular in treating complex diseases [7]. In addition, TCM treatments for some chronic diseases, such as cancer and CHB, especially when combined with western medicine, could produce incredible effects [8, 9]. TCM-syndrome, which is also called “ZHENG” in Mandarin Chinese, is the basic unit and the main perspective in TCM, which could help us understand the body homeostasis and provide guidance for the treatment of diseases [10, 11]. In some Asian countries, TCM based on syndrome has been used to diagnose and guide the treatment of CHB for many years [12]. Patients who suffer from an identical disease would be treated with different methods based on the TCM-syndromes and vice versa. Different TCM-syndromes that received the same therapy could exhibit various responses [13]. Furthermore, utilizing TCM without taking syndrome classification into account could generate serious side effects [14]. On the other hand, until now, TCM-syndromes justification still mainly relies on experienced practitioners, which may lead to ambiguity and subjectivity for one syndrome by different doctors [12]. However, in reality, modern medicine has been closely combined with TCM, such as the biochemical indicators from modern clinical examination have been widely used in TCM diagnosis, and that is why patients often receive a biochemical indicator inspection report in a TCM clinic. These biochemical indicators could reflect the physical state more objectively than TCM-syndrome diagnosed by experienced practitioners. Therefore, establishing the relationship between these biochemical indicators with TCM-syndromes will make great significance for the development of TCM. Fortunately, considerable achievements have been made in this field [15].

Spleen-stomach dampness-heat syndrome (SSDHS) and liver-depression spleen-deficiency syndrome (LDSDS) are two common TCM-syndromes for CHB. A large sample investigation conducted by our team found that among 1260 CHB patients, 16.7% were SSDHS and 8.8% were LDSDS [16]. Thus, looking for effective biochemical indicators to differentiate SSDHS and LDSDS could be quite helpful for clinical considerations. In addition, the previous mRNA screening for the CHB two TCM-syndromes (SSDHS and LDSDS) in our laboratory found that there were 9 differentially expressed genes between the two syndromes. Among them, the expression of HIST2H2BE (H2BE) was the most significant one [17]. Studies found that the expression levels of histone could participate in many cellular processes, such as DNA replication, repair, recombination, transcriptional regulation, etc. [18, 19]. Further studies confirmed that histone fragments encoded by H2BE could act on the immune dominant epitopes in Th cells and then further stimulate the proliferation, differentiation, and IL-2 release of Th cells [20]. Under the stimulation of hepatitis B antigen, Th cells then mainly differentiate into two subgroups, Th1 and Th2. Th1 mainly participates in the cellular immune response by secreting interleukin-2 (IL-2) and INF-*γ*, and Th2 is mainly involved in the humoral immune response by secreting interleukin-4 (IL-4), interleukin-10 (IL-10) [21]. Thus, the equilibrium state of Th1/Th2 is considered as an important effective factor of hepatitis B chronicity [22].

Collectively, we suggested that the differential expression of H2BE in SSDHS and LDSDS could further lead to the ratio of Th1/Th2 change and then making SSDHS and LDSDS possess the different levels of cytokines. In order to investigate whether there is any relationship between CHB TCM-syndromes and cytokines, in this paper, we chose IL-2, IL-4, IL-10, and INF-*γ* as the research objects and examined the expression level of H2BE, IL-2, IL-4, IL-10, and INF-*γ*. Furthermore, the correlation between TCM-syndromes and the specific cytokines was analyzed. Our results could provide more details for the causes of the same disease with different TCM-syndromes and a reference for the further development of biochemical indicators of SSDHS and LDSDS syndromes.

## 2. Materials and Methods

### 2.1. Participants

A total of 31 CHB patients were enrolled from August 2018 to February 2019 in the Outpatient Department of Hepatology, General Hospital of Chengdu Military Region. Among the 31 CHB patients, there were 11 SSDHS CHB patients, 11 LDSDS, and 9 healthy volunteers (control group, CG). All the enrolled CHB patients underwent rigorous screening according to the selection criteria. The inclusion criteria were as follows:

1. CHB patients' TCM-syndrome was differentiated by Chinese physicians with the title of associate professor or above
2. The age of these cases who met the selection criteria in this study was between 18 and 65, male or female
3. In addition, according to the diagnostic criteria in the 2015 guidelines for the prevention and treatment of chronic hepatitis B in China, CHB patients in this study were HBsAg or HBV-DNA positive for at least six months

The details of the samples are listed in Table 1. The TCM-syndrome types were identified according to the TCM-syndrome standards of chronic hepatitis B established by the Chinese Society of Traditional Chinese Medicine, 2017 [23]. This study was approved by the Medical Ethics Committee of Affiliated Hospital of Chengdu University of Traditional Chinese Medicine, China.

### 2.2. RNA Isolation and qRT-PCR Analysis

All the blood samples were collected from the participants' peripheral venous blood, and the samples were frozen in liquid nitrogen immediately and then stored at −80°C. Total RNAs were extracted by using TRIzol® Reagent (Life Technologies, USA) according to the manufacturer's protocol. The primers used for qRT-PCR analyses are listed in Table 2. CDNAs were synthesized by using the SuperScript™ III First-Strand Synthesis System (Life Technologies, USA). SYBR-based qRT-PCR (SYBR® Green Master Mix, Life Technologies, USA) were performed on a step-one real-time system (ABI company) by using the following reaction conditions: predenaturation at 95°C for 2 min, followed by 40 cycles of 95°C for 10 s and 60°C for 10 s, 40 cycles. Each sample was performed in three technical triplicates.

### 2.3. Serum Cytokine Measurements

Serum samples from 11 SSDHS, 11 LDSDS, and 9 healthy volunteers were collected by centrifuging at 3500 r/min, 4°C for 10 min, and stored at −80°C. Four Th related cytokines (IL-2, IL-4, IL-10, INF-*γ*) were detected using human cytokines ELISA kits (Life Technologies, USA) based on the manufacturer's protocol. The optical density values of the four cytokines were determined by using a Labsystems Multiskan MS Reader. The concentration of each cytokine was calculated using a standard curve.

### 2.4. The Protein-Protein Interaction Networks Construction

The protein-protein interaction networks between H2BE and the four cytokines were constructed by using the GeneMANIA database (http://genemania.org/). Briefly, input the gene name of HIST2H2BE, IL-2, IL-4, and IL-10, respectively, and then click the search button to construct the networks.

### 2.5. Statistical Analysis

Data of qRT-PCR were processed using Microsoft 2010 based on the 2^−ΔΔCt^ method and the CG group was set as the control. The significance between the experimental groups (LDSDS, SSDHS) and control group (CG) was detected by using the one-way ANOVA method via the SPSS 24.0. In addition, the expression levels of the four cytokines were also analyzed using SPSS 24.0. The standards for the potential marker selection are that the potential marker which could not only distinguish the two syndromes but also could distinguish the syndromes from the healthy group (the significance condition is at least *P* value <0.05).

## 3. Results

### 3.1. Patient Characteristics

A total of 31 participants were enrolled in this study, including 11 LDSDS, 11 SSDHS, and 9 healthy subjects. Among the 11 LDSDS, 5 were males and 6 were females; among the 11 SSDHS, 7 were males and 4 were females; among the 9 healthy participants, there were 4 males and 5 females (Table 1). There was no significant difference in age at baseline (Table 1).

### 3.2. Cytokines Expression Level Analysis

To ascertain the expression levels of cytokines between CHB patients and healthy subjects, and also to identify the differently expressed cytokines between the LDSDS and SSDHS, the expression levels of the four cytokines were detected by ELISA. The results showed that the expression levels of IL-2 were significantly increased (*P* < 0.01) in LDSDS and SSDHS compared with the healthy group; however, there is no significant difference observed (*P* > 0.05) between LDSDS and SSDHS (Table 3). For IL-4, both LDSDS and SSDHS exhibited increased levels (*P* < 0.05) compared with the healthy group (Table 3). Significant increase of IL-10 was observed only in LDSDS, but not in SSDHS (*P* < 0.01) when compared with the healthy group, and there is a statistical difference (*P* < 0.05) between LDSDS and SSDHS. Finally, the expression levels of INF-*γ* in different groups were analyzed. Results showed that the expression levels of INF-*γ* in LDSDS and SSDHS showed a dramatic increase (*P* < 0.05) compared with the healthy group. Notably, the expression level of INF-*γ* in LDSDS was even increased compared with SSDHS (*P* < 0.01) (Table 3). Collectively, what attracts us most is that among the four cytokines, only IL-4 and INF-*γ* showed statistical differences not only between these two syndromes but also between the syndromes and healthy group, suggesting that these two cytokines may serve as the potential biochemical indicators of the TCM-syndromes of CHB, but these need to be further studied.

### 3.3. Analysis of H2BE Gene Expression Level

**Figure 1:**
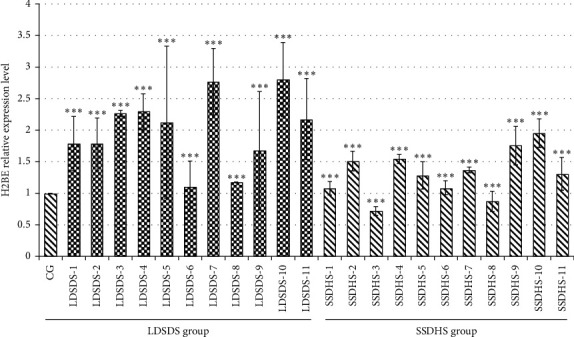
qRT-PCR validation of the H2BE gene in the three groups (LDSDS, SSDHS, and healthy group). The *X*-axis represents the samples studied in this study; the *Y*-axis represents the relative expression levels of genes. U6b gene was selected by our laboratory previously, which was used as a control. CG represents the healthy group. All data are from three technical repeats (*n* = 3). ^∗∗∗^represents the significance level of the one-way ANOVA test, *P*=0.001. Error bars denote |S|D.

In order to illuminate the expression levels of H2BE in LDSDS, SSDHS, and the healthy group, we performed the qRT-PCR. QRT-PCR results showed that LDSDS-10 exhibited the highest expression level in the LDSDS group, while the lowest expression level was LDSDS-6. In the SSDHS group, SSDHS-10 showed the highest expression level and SSDHS-3 exhibited the lowest. Interestingly, the expression levels of H2BE in LDSDS were generally higher than that in SSDHS, which was consistent with the cytokines results (Figure 1). Moreover, when compared with the CG group, the expression levels of the H2BE in most of the CHB patients showed a trend of increase, suggesting that the expression level of H2BE may be related to the formation of TCM-syndromes to some extent, but this needs to be further studied in the following research.

### 3.4. Correlation Analysis between H2BE and the Cytokines

**Figure 2:**
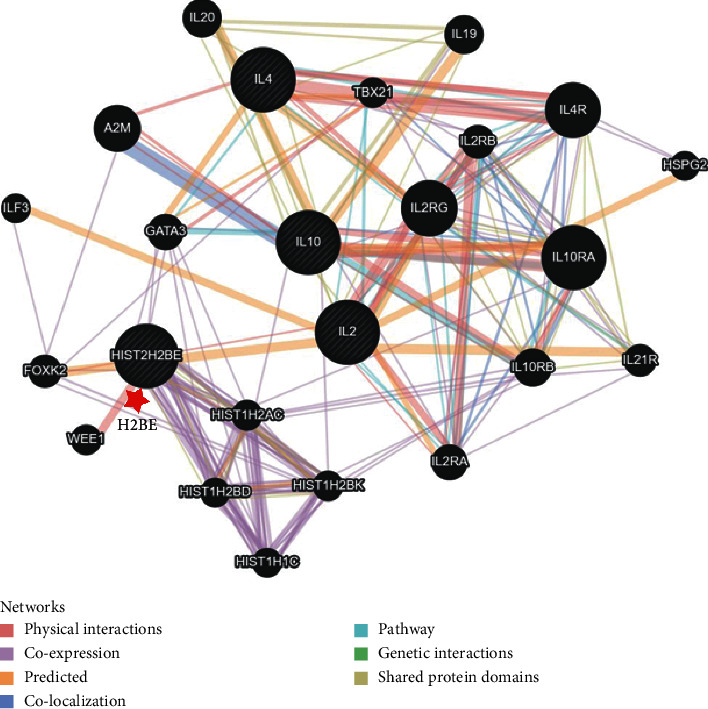
The protein-protein interaction networks between H2BE and the four cytokines. The networks between H2BE and the four cytokines were constructed by using the GeneMANIA database.

**Figure 3:**
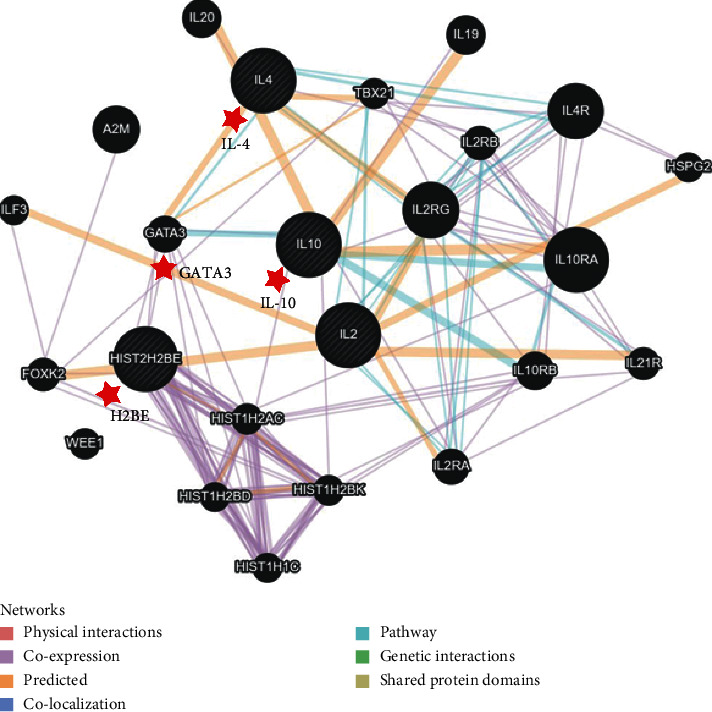
The major relationships with H2BE gene in the cytokines expression regulation. The networks between H2BE and the four cytokines were constructed by using the GeneMANIA database.

In order to further elucidate how H2BE works on the cytokines and also mine the latent genes which participate in the expression regulation of H2BE on the cytokines, the protein-protein interaction networks between H2BE and the four cytokines were constructed. The results demonstrated that H2BE, IL-2, IL-4, IL-10, and some other related molecules were correlated mainly through physical interaction (67.64%), coexpression (13.5%), and signaling pathway (4.35%) (Figure 2). Furthermore, after analyzing the major relationships in the network, we found that H2BE was indirectly associated with IL-4 and IL-10, and H2BE regulated the expression level of cytokines mainly through GATA3 (Figure 3). That is, the GATA3 gene acts as a bridge between H2BE and cytokines and also showed a coexpression pattern with H2BE, IL-4, and IL-10. These remind us that GATA3 may play an important role in the regulation of H2BE on cytokines expression, but this needs to be further researched.

## 4. Discussion

Hepatitis B is a hidden killer. People infected with a virus for decades may show only mild symptoms, which could be easily ignored and may ultimately develop into liver cirrhosis or even liver cancer [24]. So far, the direct-acting antiviral agents (DAAs) of western medicine still could not eliminate the virus completely [25]. Therefore, pretreatment is of great value in the treatment of CHB and liver cirrhosis. TCM has been widely used in CHB pretreatment since its unique advantages, such as low toxicity side effect, good curative effect, etc. [26]. TCM syndrome plays an important role in TCM treatment, and the same disease with different TCM syndromes in western medicine could receive different treatments in TCM [11]. However, due to the fact that TCM-syndrome is overrelying on experience, this greatly limits its application worldwide. Thus, excavating available biomarkers for TCM-syndrome diagnosis and elucidating the underline mechanisms of TCM-syndrome could greatly help the application of TCM all over the world.

Previous studies found that the imbalances of Th1/Th2 cells and their cytokines are important causes of CHB [27]. However, Th1 and Th2 levels are strongly associated with the cytokine levels elaborated above [21]. For different CHB TCM-syndromes, as the TCM-syndrome changes, the immune responses may also be changed due to the ratios of different cytokines are also changed. In this study, we found that the expression levels of almost all the examined cytokines were upregulated compared with the healthy group. Among the four cytokines, IL-4 and INF-*γ* showed statistical significance not only between the syndromes but also among the syndromes and healthy group, and these two cytokines may have potential as the biochemical indicators of the two TCM-syndromes. In addition, the results also demonstrated that almost all the cytokines were expressed much higher in LDSDS than in SSDHS, and this suggested that the immune response may be more intense in LDSDS than in SSDHS, and this result was also consistent with Lu et al. (2019) [11].

Histone is involved in many cellular processes such as transcriptional regulation, DNA replication, RNA splicing, etc., since the DNA is wrapped and interacts directly with histone proteins [28]. However, at present, a myriad of studies on histone mainly focus on histone modification, such as methylation, acetylation, phosphorylation, etc., rather than the effects generated by expression level changes [18, 29]. In yeast cells, researchers found that overexpression histone H4 could suppress SIR2-induced inviability, while overexpression H3 did not [30]. Singh et al. [19] found that overexpression histone H2A resulted in a significant decrease in susceptibility towards drugs currently in use in Leishmania parasites, and thus pointing to its role in drug resistance. For H2BE, a study on breast cancer found that overexpression or downregulation of H2BE could both lead to the decreased proliferation in breast cancer cell lines [31]. Furthermore, the expression level of H2BE was also associated with the cell function. That is, different cell types exhibit different expression levels of H2BE [32]. These indicated that H2BE might play an important role in regulating the physiological function of cells. Interestingly, in this study, we found that the expression levels of H2BE in LDSDS were basically higher than that in SSDHS and showed an increasing tendency in CHB patients compared with the healthy group.

In the nucleosome, H2BE is mostly exposed to the surface of the nucleosome compared with other nucleosome proteins [33, 34]. Hao [20] found that the subunit from 14th-28th amino acid of H2B was the dominant epitope that could interact with Th cells and stimulate more cytokines secreted. In this study, we have also investigated the protein-protein interaction networks between H2BE and the four cytokines, and the results displayed that GATA3 protein acts as a bridge between H2BE and cytokines and also showed a coexpression pattern with H2BE, IL-4, and IL-10. A myriad of studies reported that GATA3 could be expressed in large quantities in immune cells, such as T lymphocytes, NK cells, NKT cells, etc. [35–37]. Chen et al. [38] found that overexpression GATA3 could affect cytokine secretions of Th1 and Th2. Thus, we suggested that one other possible way for the expression regulation of Th related cytokines by H2BE is through the direct or indirect interaction between H2BE and GATA3, among which GATA3 may be the intermediate node, with coexpression and other related pathways.

**Figure 4:**
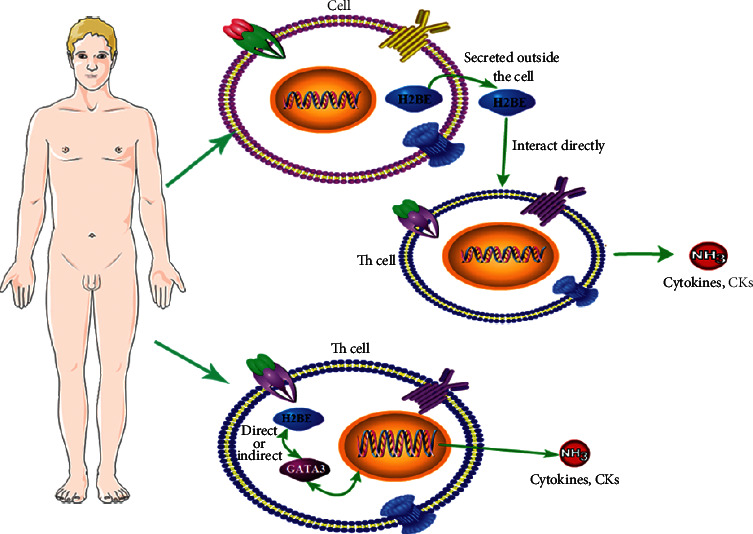
The possible regulatory pathways of H2BE involved in the secretions of cytokines.

In summary, we believe that H2BE regulates cytokine secretions via the following two ways, (1) H2BE could interact with Th cells directly through a dominant epitope way, and (2) the other way is by regulating the GATA3 gene directly or indirectly and then further regulating the cytokine secretions (Figure 4). However, further studies are needed to clarify the relative regulation mechanisms of H2BE.

## Figures and Tables

**Table 1:** Baseline comparison of the clinical characteristics among the groups.

Group	Number of cases	Gender^a^		Age^b^$\left(\bar{x}\pm s\right)$
		Male	Female	
LDSDS	11	5 (45.5%)	6 (54.5%)	39.27 ± 8.661^c^
SSDHS	11	7 (63.6%)	4 (36.4%)	36.00 ± 12.578^c^
CG^d^	9	4 (44.4%)	5 (55.6%)	33.56 ± 8.00^c^
Total	31	16 (57.69%)	15 (42.31%)	—

^a^There was no significant difference in gender composition among the three groups by the chi-square test (*P* > 0.05). ^b^There was no significant difference in age composition among the three groups by one-way analysis of variance (*P* > 0.05). ^c^mean ± standard deviation. ^d^Healthy group (control group).

**Table 2:** The primer pairs for qRT-PCR validation.

Primers	Sequence (5'-3')
H2BE-F	agtggctgagttcggctgtc
H2BE-R	gctgccaagcgtcagtcata
U6b-F	ggcagtcgaccgacgaata
U6b-R	cgtgaaagaccgcagcaa

**Table 3:** Expression levels of the four cytokines detected with ELISA.

Cytokines	LDSDS	SSDHS	CG
IL-2	133.451 ± 20.078^※※^	121.870 ± 21.934^※※^	76.308 ± 14.061^a^
IL-4	68.194 ± 10.387^※※##^	50.432 ± 7.936^※^	39.618 ± 7.953^a^
IL-10	123.728 ± 26.204^※※#^	93.452 ± 13.689	81.416 ± 10.387^a^
INF-*ɣ*	159.037 ± 33.737^※※##^	106.233 ± 23.602^※^	80.097 ± 16.610^a^

^a^mean ± standard deviation. ^※^The difference was statistically significant compared with the healthy group (*P* value <0.05). ^※※^The difference was statistically significant compared with the healthy group (*P* value <0.01). ^#^The difference was statistically significant compared with the SSDHS group (*P* value <0.05). ^##^The difference was statistically significant compared with the SSDHS group (*P* value <0.01).

## Data Availability

The datasets used and/or analyzed during the present study are available from the corresponding author upon reasonable request.
